# Farewell Editorial

**DOI:** 10.1038/s41439-020-0093-3

**Published:** 2020-03-19

**Authors:** Katsushi Tokunaga

**Affiliations:** Genome Medical Science Project-Toyama, National Center for Global Health and Medicine, (The University of Tokyo), Tokyo, Japan

At the end of 2019, I retired after 6 years as the founding Editor-in-Chief for *Human Genome Variation (HGV)*. Before this, I had been Editor-in-Chief for the *Journal of Human Genetics*, the official journal of The Japan Society of Human Genetics (JSHG) for 6 years. During this time, I found myself having to reject many submitted manuscripts. However, this was often with regret as many of those manuscripts actually contained results which were worth sharing among both researchers and clinicians. It was also clear that the rapid development of next-generation sequencers would lead to an extraordinary increase in sequence and variation data. This led me to the idea of a new open-access journal containing articles and reports to allow data sharing about variation and variability in the human genome, and the implications and future impacts for the study of human genomics. Accordingly, in 2012, with support from JSHG and Nature Publishing Group (now Springer Nature), I proposed a new open-access, online-only journal and fortunately obtained a 5-year grant from the Japan Society for the Promotion of Science in order to successfully launch *HGV* as the second official journal of the JSHG in 2014.

An important and innovative feature of *HGV* is “Data Reports”, short reports about human genome variation and variability which describe disease-causing variation and/or their frequencies in different human populations. A further important feature of *HGV* is a curated database of the underlying data from “Data Reports”, which has grown into an important resource for the genomics and clinical genetics communities. In 2019, *HGV* started “Software Reports”, which provide useful software tools for human genome data analysis. I believe these innovations have become valued in the relevant research communities. Of course, *HGV* also publishes Articles and Reviews on the relevant topics in human genome studies.

I would like to thank all the Associate Editors and Editorial Board members, and the hundreds of reviewers and authors for their enormous help in the publishing and development of *HGV*. I also would like to acknowledge staff in Springer Nature, Masayo Kobayashi, Emi Sakuyama, Yoko Shintani, and Ludivine Allagnat. I believe the steady growth of the journal promotes data sharing and open science in our research fields.

I am happy that Prof. Issei Imoto, MD, PhD, has agreed to be the new Editor-in-Chief. Under his new leadership and with his enthusiasm, *HGV* will show further improvement and innovation, and gain wider recognition.

My deepest thanks to everyone!

Sayonara!

